# A Rare Case of a Meckel's Diverticulum Complicated by a Mesodiverticular Band

**DOI:** 10.7759/cureus.67364

**Published:** 2024-08-21

**Authors:** Abhishek Shenoy, Digvijay Jadhav, Rishabh Gandhi, Rushabh A Parekh

**Affiliations:** 1 General Surgery, Dr. D. Y. Patil Medical College, Hospital and Research Centre, Pune, IND

**Keywords:** meckel's diverticulum, complicated meckel's diverticulum, internal hernia, intestinal obstruction, mesodiverticular band

## Abstract

A Meckel's diverticulum is a true congenital diverticulum arising from the ileum, approximately 2 feet from the ileocaecal junction. Named after Johann Meckel, who first described its embryological origins, the anomaly remains asymptomatic for most. Uncommonly, it is found to be the cause of serious complications such as interstitial obstruction and/or gangrene, bowel perforation, and, in rare cases, internal bowel herniations. A mesodiverticular band is a congenital fibrous band connecting the Meckel's diverticulum to its own mesentery, predisposing it to complications. Both conditions arise from a failure of regression of the vitellointestinal duct and its feeding artery. The presence of a mesodiverticular band significantly raises the possibility of complications, especially those of internal herniation and subsequent bowel obstruction. Detection of a Meckel's diverticulum is challenging in routine investigations such as contrast-enhanced computed tomography, and scintigraphy with Tc99 is required. The availability of such scans is limited, and their use is further difficult in emergent situations such as intestinal obstruction. This condition is, therefore, more frequently detected at laparotomies. Herein, we report a case of intestinal obstruction in a young female who presented to our emergency room with an acute abdomen and was found to have a mesodiverticular band causing internal herniation and subsequent obstruction.

## Introduction

Meckel's diverticulum (MD) is the most commonly encountered anomaly of the gastrointestinal tract. Arising approximately 100 feet from the ileocaecal junction, it is an embryonal remnant of the vitellointestinal duct. Although in most cases the condition remains asymptomatic, it can present infrequently in the pediatric population with serious complications, the most emergent of which is small intestinal obstruction.

Complications in adults are rare [1]. According to estimates reported in a study, there is a 4% lifetime risk of complications from MD. Approximately 80% to 95% of cases are found to be asymptomatic. The following complications can occur: painless lower gastrointestinal bleeding (4%), intestinal obstruction (6%), volvulus, herniation, or entrapment of a bowel loop through a defect in the diverticular mesentery around a fibrous band. Other complications include incarceration within a hernia sac, chronic Meckel's diverticulitis, foreign bodies, or neoplasms [2].

Persistence of the omphalomesenteric duct artery results in a fibrous band found in 1%-4% of the population [3]. It extends between the mesentery of the MD and its tip. This is known as a mesodiverticular band (MDB) and increases the risk of serious complications such as internal herniation of bowel loops through the opening thus created. This may result in obstruction, strangulation, and gangrene of the bowel loop that has herniated through the space created by such a band. In rare situations, adhesions to the lateral pelvic wall may also be seen, further increasing the chances of complications.

The first report of a case of small bowel strangulation secondary to MDB was put forward by Estricht et al. (1834), which was in an adult female. Manning and McLaughlin (1947) [4] have shown that bowel strangulation due to MDB has a near 100% mortality rate.

The case reported here is of a 36-year-old female who was brought to the emergency room with an acute abdomen, which, on exploration, revealed an MD with twisting and entrapment under an MDB causing obstruction.

## Case presentation

A 36-year-old female was brought to the emergency department with severe, diffuse pain in her abdomen for eight days, which was sudden in onset. This was associated with constipation and progressive distension of her abdomen six days before presentation. She also had multiple bouts of vomiting, approximately seven to eight episodes per day since the pain started eight days ago, containing ingested food mixed with watery fluids. She also reported reduced appetite, malaise, and weakness since the onset of the symptoms.

There was no history of fever, trauma, or diarrhea. She had three cesarean section deliveries, the most recent of which was three years ago. She reported no similar complaints in the past and had no comorbid conditions.

On initial clinical evaluation, the patient had tachycardia (110/min) and hypotension (80/60 mmHg). She appeared pale and was febrile (101.2 °F). The abdomen was symmetrically distended with a healthy scar from prior cesarean sections. A small (~2x2 cm) partially reducible umbilical hernia was also noted. On palpation, there was diffuse tenderness in all quadrants with no palpable organomegaly and no lump or mass. On auscultation, bowel sounds were absent in all quadrants. There was no rebound tenderness. A rectal examination revealed a collapsed rectum with no stools.

**Figure 1:**
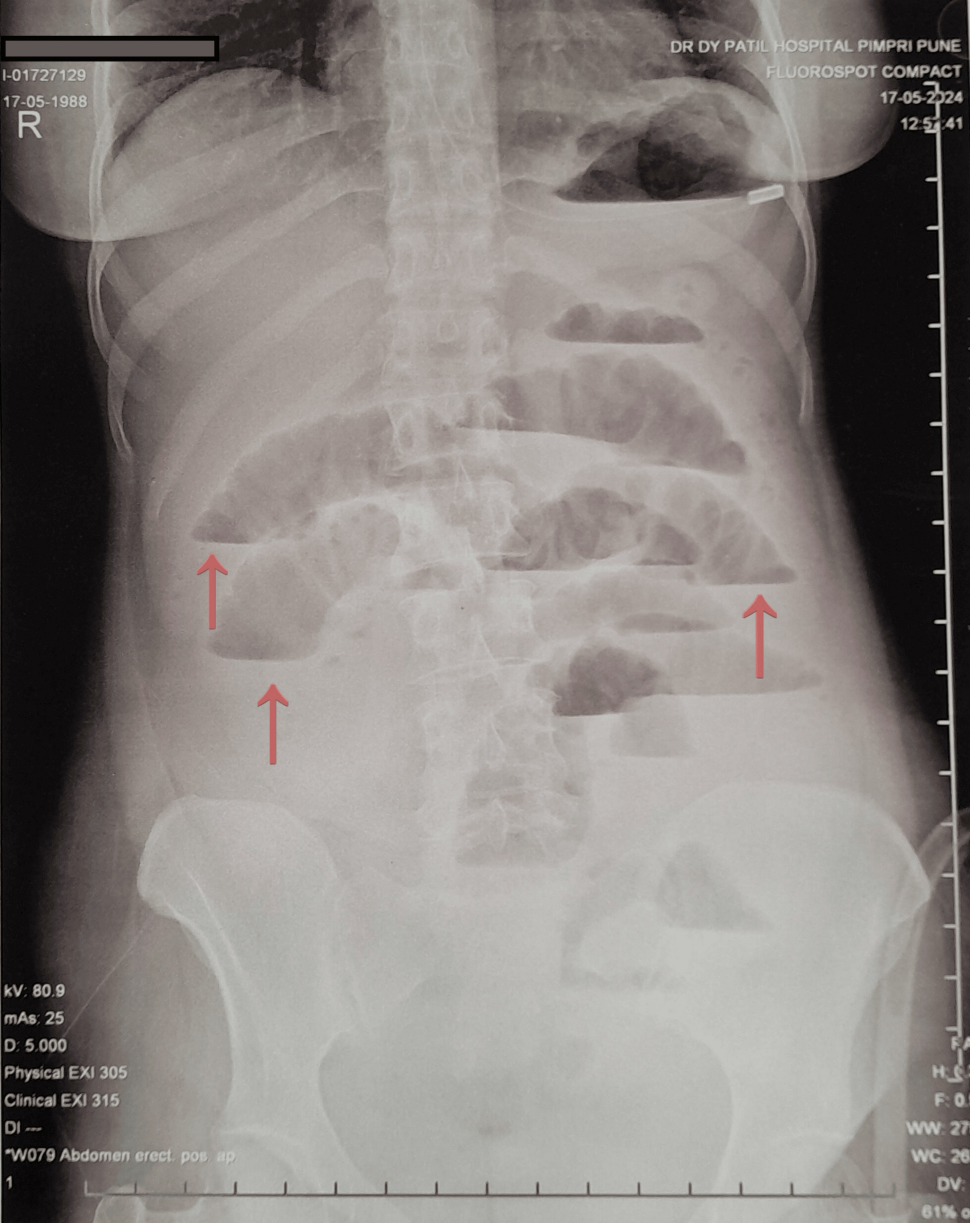
Erect abdominal X-ray showing multiple air-fluid levels (red arrows), suggestive of small bowel obstruction.

The patient was stabilized with intravenous fluid boluses. A nasogastric tube was inserted, and approximately 300 mL of feculent and bilious fluid was drained over the first hour of admission. An erect abdomen X-ray showed multiple air fluid levels with dilated bowel loops, suggestive of small bowel obstruction (Figure 1).

**Figure 2:**
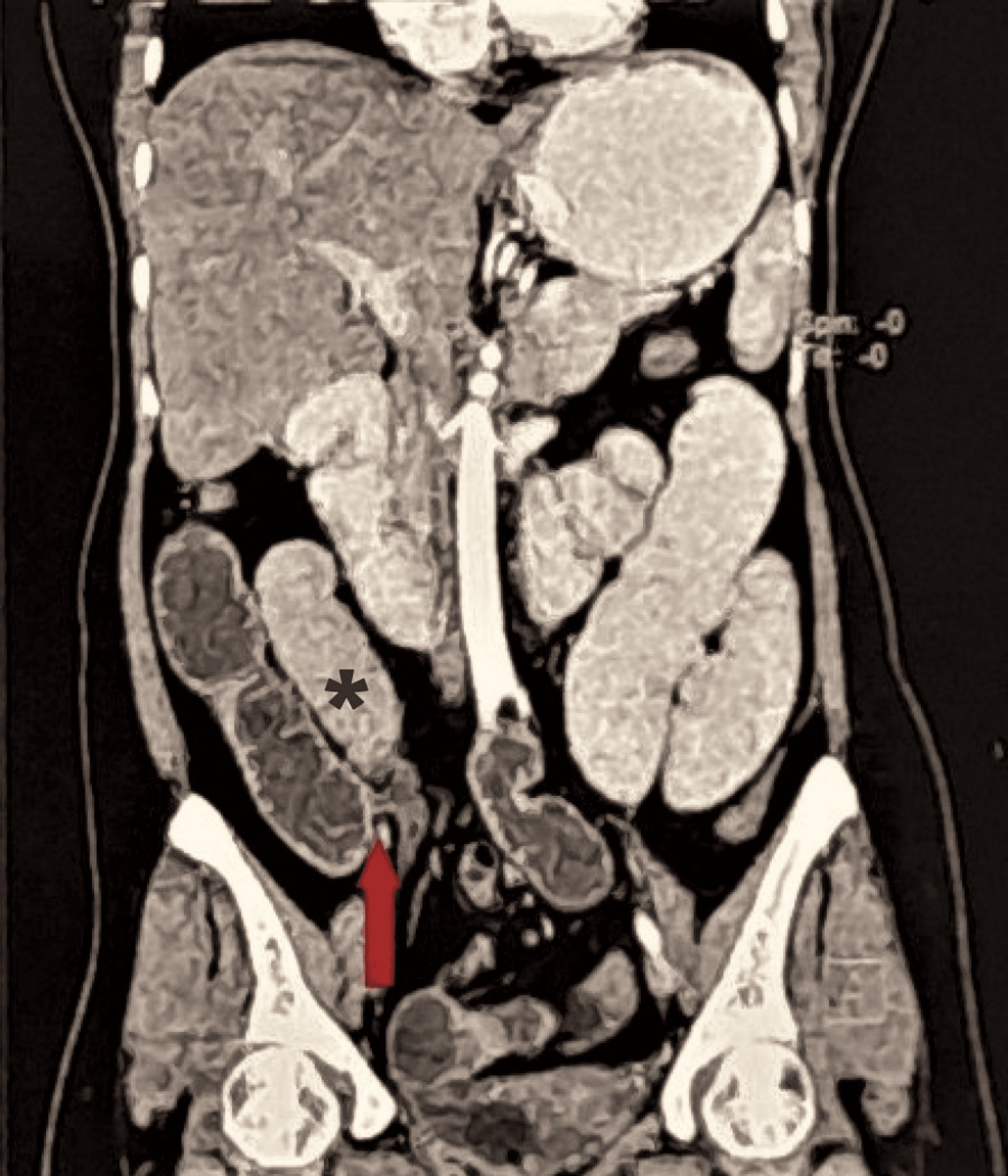
CT of the abdomen and pelvis showing bowel constriction (point of transition with kinking of the bowel) (red arrow) in the right paramedian region. The dilated bowel loop proximal to the constriction (black asterisk) is particularly noteworthy.

The patient was further evaluated. A contrast-enhanced computed tomography (CECT) of the abdomen was done, which showed dilation of the small bowel loops involving the duodenum, jejunum, and ileum with a maximum caliber of 3.7 cm. A transition zone was noted in the hypogastric region in the midline with abrupt narrowing, kinking, and twisting of bowel loops and the mesentery. The rest of the bowel loops distal to this point appeared to have collapsed (Figure 2).

**Figure 3:**
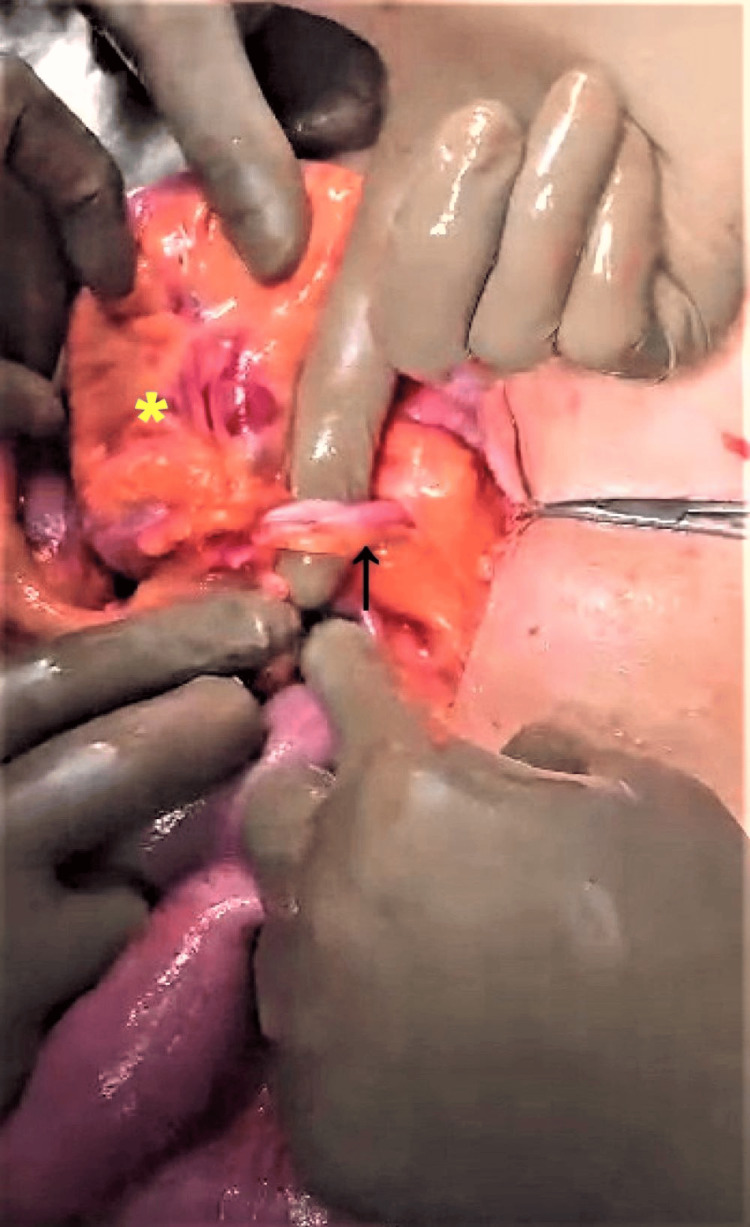
Intraoperative picture showing the mesodiverticular band (black arrow) with a gloved finger in space created by the band. A small bowel loop is seen incarcerated through it (yellow asterisk).

**Figure 4:**
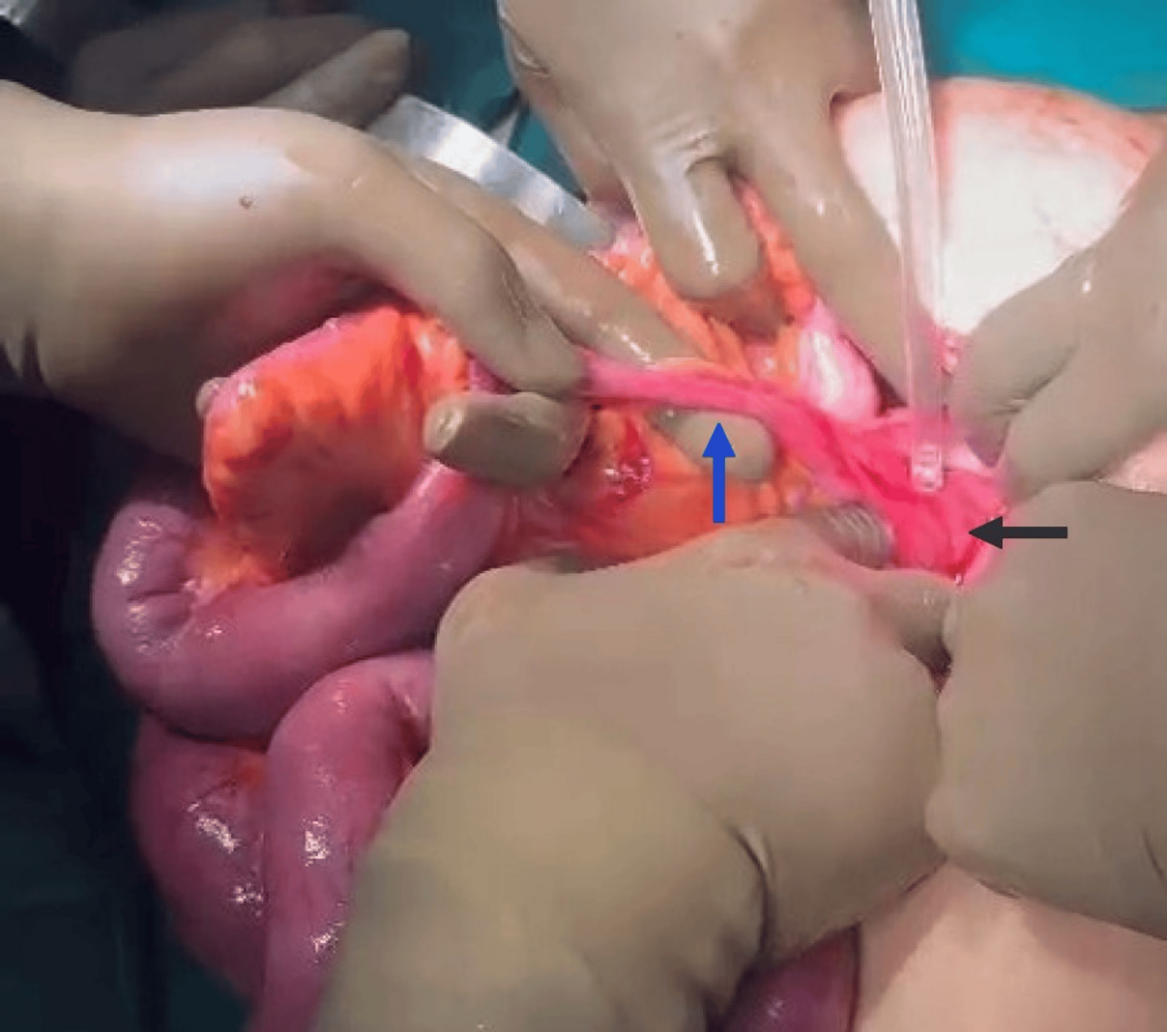
Intraoperative picture showing mesodiverticular band (blue arrow) extending to and adhered with the lateral abdominal wall (black arrow).

The patient underwent an exploratory laparotomy, which revealed dilated bowel loops twisted under a band of fibrous tissue. (Figure 3). On further exploration, the release of adhesions and untwisting of the bowel loops was performed. An MD was discovered, with a constriction 2 cm proximal to the base. The tip of the diverticulum was found within an MDB, extending from the diverticulum to the lateral abdominal wall. It was also found to have dense adhesions to the round ligament inferiorly on the right side (Figure 4).

**Figure 5:**
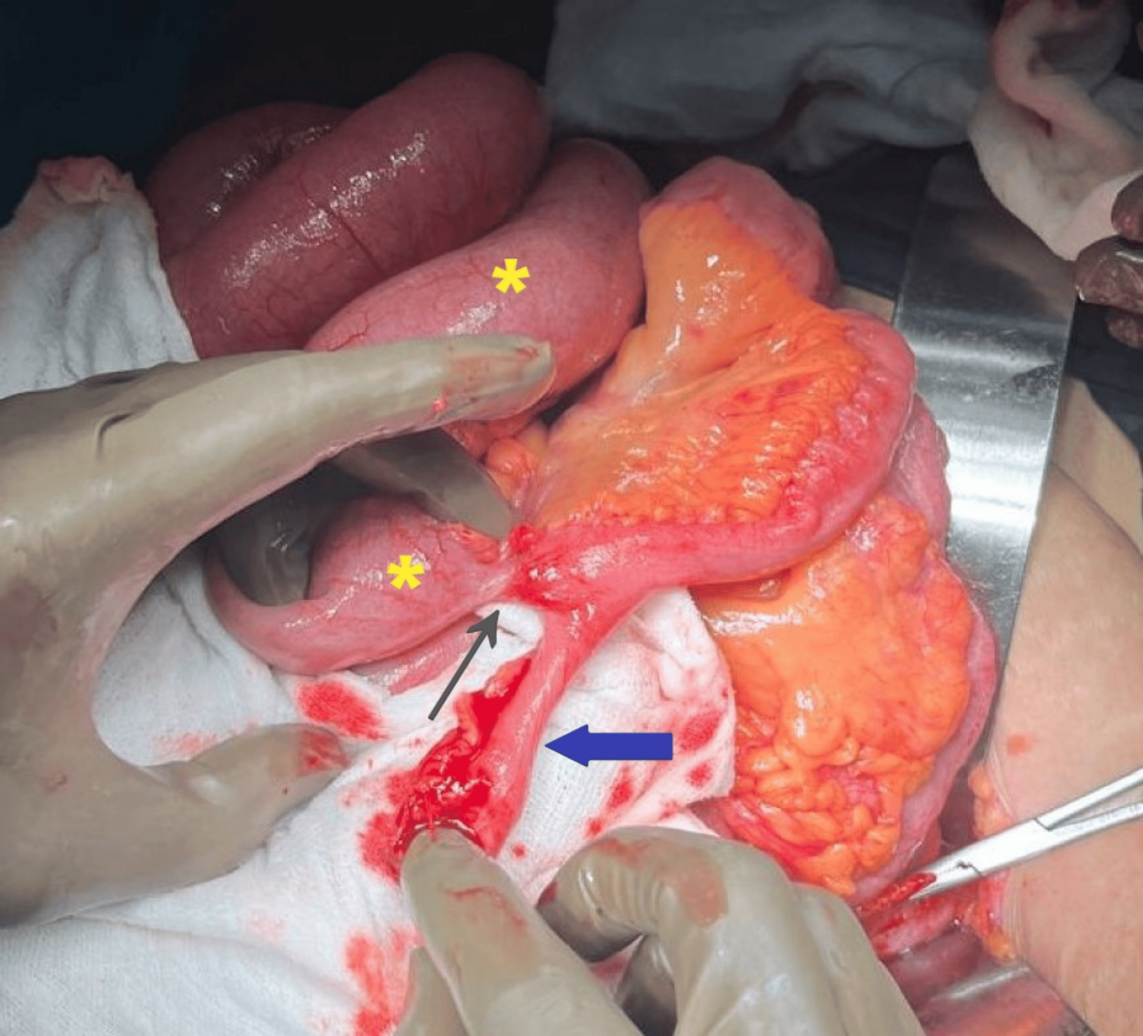
Intraoperative picture showing the Meckel's diverticulum (blue arrow) with a constriction immediately proximal to the base (black arrow). Note the proximally dilated bowel loop to the left of the picture (yellow asterisks), demonstrating the point of transition at the site of constriction.

After separating the adhesions with the round ligament, the MDB was divided between clamps, 2 cm distal to the palpable tip of the diverticulum (Figure 5). This was followed by resection of an approximately 20 cm segment of the ileum, including the MD and the constriction band. An anastomosis was performed, maintaining bowel continuity.

Drains were placed and secured, and the abdomen was closed in layers. The patient recovered well postoperatively and was discharged on the sixth postoperative day after the drains were removed.

**Figure 6:**
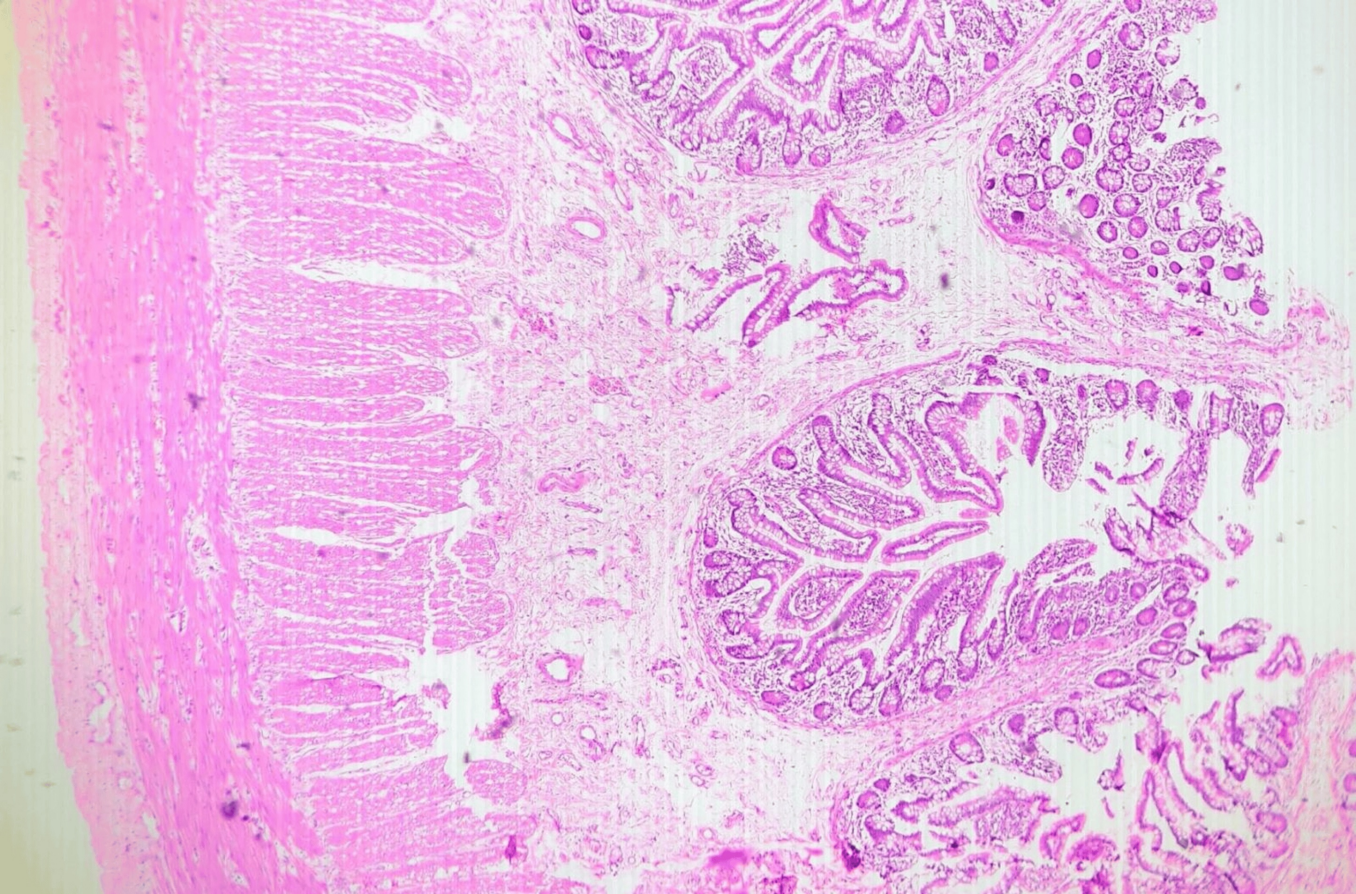
Histopathology image of the tip of the diverticulum, showing chronic inflammation of the ileal mucosa. Hematoxylin and eosin-stained section through the tip of the Meckel's diverticulum (4× magnification)

The specimen's histopathology revealed an MD with normal ileal mucosa and chronic inflammation. The section showed no evidence of heterotopic mucosa (Figure 6).

## Discussion

Embryology of MD and MDB

During embryonic life, the omphalomesenteric duct connects the yolk sac to the developing midgut. The duct stays connected to the developing placenta through the umbilicus and umbilical cord while the developing gut lengthens and rotates. The median umbilical ligament is formed by the duct's natural involution over its complete length, from the umbilical opening to the ileum. An MD develops when this natural regression fails at the ileal (proximal) end of the duct. It is the most frequently seen anomaly of the GI tract and is found in approximately 2% of the population. The majority of such diverticula are asymptomatic [1].

MDB

In rare situations, an MDB is found to be attached to the diverticulum. Embryologically, it is a remnant of the vitelline artery that supplies MD. It is seen as a fibrous band between the tip of the diverticulum and its mesentery. This band creates an opening through which any adjacent part of the small bowel and, rarely, the MD may herniate, causing constriction, edema, and eventually obstruction.

Complications

Complications are described only in up to 6% of cases of MD, which most commonly include hemorrhage, intestinal obstruction, diverticulitis, and perforation. The following factors were found to predispose the patient to complications: (1) age less than 50 years; (2) male sex; (3) a diverticulum longer than 2 cm; and (4) the presence of ectopic mucosa within (Table 1).

**Table 1:** Complications associated with Meckel's diverticulum. Reference: [3]

Complications	Incidence in Percentage	Children	Adults	Cause
Hemorrhage	25-50	Most common presentation	-	Heterotopic mucosa (gastric and pancreatic); neoplasms ( carcinoid tumors, adenocarcinomas, benign mesenchymal tumors, melanoma, lymphoma, lipoma)
Intestinal obstruction	22-50	Second most common presentation	Most common presentation	Intussusception, volvulus, Littre's hernia, mesodiverticular band, stricture, tumors, fecal/meconium impaction)
Inflammatory process (diverticulitis, perforation)	~20	-	Second most common presentation	Diverticulitis: obstructions due to fecolith/foreign body or parasites; peptic ulceration of the ilea's mucosa due to ectopic gastric mucosa; diverticular torsion. Perforation: progression of diverticulitis, ulceration secondary to acid production from ectopic gastric mucosa, trauma, tumors
Tumors	0.5-1.9	-	-	Benign: lipoma, hamartoma; malignant: carcinoids, adenocarcinoma, mesenchymal tumors

Small bowel obstruction occurs in one-third of adults with an MD, making it the most frequently encountered complication. It is caused by various mechanisms, including volvulus, Littre's hernia (MD as the content of an inguinal hernia ), intussusception of an inverted MD, and internal herniation of the small bowel through the space created by the MDB.

An MD connected with the MDB is rare. When present together, they frequently cause obstruction that necessitates emergent surgery. The small bowel loops adjacent to the diverticulum herniate internally through the MDB, forming the major mechanism for bowel obstruction (as seen in the presented case). Rarely, axial torsion of the MD may occur, causing small bowel volvulus.

Complications related to the MDB may present at all ages, in contrast to the usual presentation of an MD alone seen in the pediatric population [5]. The patient evaluated here is a 36-year-old female who is an atypical age at presentation for complications resulting from MD.

In adults and children, the discovery of an MD was three times as likely in males as compared to females (72% vs. 28%). The asymptomatic versus symptomatic MD ratio was also 3:1 in children but nearly equal in adults (58% vs. 42%). The above demographics make the case presented here rare and reportable.

Diagnosis

The accurate diagnosis of an MD is challenging. An MDB complicating the condition with intestinal obstruction is associated with a high mortality risk, as seen in multiple case reports, most notably put forward by Vork et al. [6]. Complications may clinically resemble other abdominal diseases, such as acute appendicitis, colitis, inflammatory bowel disease, ureteric obstruction, ovarian torsion, or other causes of small bowel obstruction [7]. A comparative study between incidentally discovered and symptomatic cases by Bani-Hani and Shatnawi [8] reported a preoperative diagnosis in only four patients out of 28 (5.9%) from the symptomatic group. A long diverticulum with a narrow base (diameter of < or = 2 cm) was seen to have an increased risk of complications [7].

Ueberrueck et al. [9] evaluated cases diagnosed as appendicitis in particular and found that 3% of those in which the bowel was explored at appendectomy had an MD. Approximately 9% of these diverticula were complicated with diverticulitis, intussusception, obstruction, and perforation, thus highlighting the significance of exploring the bowel at all appendectomies.

The preoperative CECT scan in our case also failed to detect the presence of the diverticulum and provided information only of the obstruction, hinting vaguely at the cause being a volvulus or twisting. This is consistent with the overall incidence found in the literature of the sensitivity of CT to detect diverticulum. The Tc99 scan so often described is only valuable if there is a suspicion of a heterotopic mucosa, such as gastric or pancreatic, with a different presentation profile (melena/hemorrhage/perforation). This was not seen in our case.

Management

Resection of an MD is advisable in symptomatic cases and can be performed in various methods, depending upon the severity of insult to the bowel in continuity with the diverticulum. In most situations where the bowel is found to be viable, a wedge resection can be performed, where only the diverticulum with the bowel in immediate contact with it can be excised, followed by an ileo-ileal anastomosis.

Alternatively, if the bowel shows signs of ischemia (dusky discoloration, non-peristaltic), perforation, or gangrene, an adequate length of the affected segment may be resected, leaving behind a healthy bowel that is subsequently anastomosed. Due to the severity of the obstruction and formation of a constriction, a decision to resect the affected length of the bowel followed by anastomosis was taken and performed in the case we present here.

There is considerable debate on whether those diverticuli found incidentally at laparotomies or appendicectomies, i.e., asymptomatic diverticuli, should be excised. The standard recommendation is the excision of an incidentally discovered MD in the pediatric age group [10]. A literature review revealed no consistent guidelines recommending resection in asymptomatic MD in adults. As such, the decision to resection may be made on a case-by-case basis, depending on the viability of the bowel at laparotomy and the complications encountered.

## Conclusions

MD causing small bowel obstruction is a known entity. The presence of an MDB that causes twisting and constriction leading to obstruction in a young female is rare. The presence of adhesions post-multiple cesarian section deliveries increased the risk of adhesions causing obstruction in the case presented here. As such, this should be highly suspected in all patients presenting with an acute abdomen with clinical and radiological findings characteristic of small bowel obstruction. In such cases, any exploration of the abdomen should include a complete bowel examination for the MD and the MDB. Surgical management can be decided on a case-by-case basis, depending on the intraoperative findings at laparotomy and the detection of the diverticulum and the MDB when present.

## References

[1] Tavakkoli A, Ashley SW (2015). Small intestine. Schwartz's Principles of Surgery, 10th Edition.

[2] Dutta G, Chowdhury AS, Panda M (2009). Band of cacophony - abdominal catastrophe caused by the fibrous band of Meckel's diverticulum: a case report. Cases J.

[3] Serdar K, Hakan B, Kemal K (2013). Mesodiverticular band of Meckel's diverticulum as a rare cause of small bowel obstruction: case report and review of the literature. Viszeralmedizin.

[4] Manning VR, McLaughlin EF (1947). Persistent omphalomesenteric (vitelline) artery causing intestinal obstruction and gangrene of Meckel's diverticulum. Ann Surg.

[5] Vork JC, Kristensen IB (2003). Meckel's diverticulum and intestinal obstruction—report of a fatal case. Forensic Sci Int.

[6] Bamarni S, HungFong S (2021). Systematic review of mesodiverticular band: a rare cause of small bowel strangulation and hemoperitoneum in adults. Int J Surg Short Rep.

[7] Cartanese C, Petitti T, Marinelli E, Pignatelli A, Martignetti D, Zuccarino M, Ferrozzi L (2011). Intestinal obstruction caused by torsed gangrenous Meckel's diverticulum encircling terminal ileum. World J Gastrointest Surg.

[8] Bani-Hani KE, Shatnawi NJ (2004). Meckel's diverticulum: comparison of incidental and symptomatic cases. World J Surg.

[9] Ueberrueck T, Meyer L, Koch A, Hinkel M, Kube R, Gastinger I (2005). The significance of Meckel's diverticulum in appendicitis—a retrospective analysis of 233 cases. World J Surg.

[10] Blouhos K, Boulas KA, Tsalis K (2018). Meckel's diverticulum in adults: surgical concerns. Front Surg.

